# A Stacking Ensemble Learning Framework for Genomic Prediction

**DOI:** 10.3389/fgene.2021.600040

**Published:** 2021-03-04

**Authors:** Mang Liang, Tianpeng Chang, Bingxing An, Xinghai Duan, Lili Du, Xiaoqiao Wang, Jian Miao, Lingyang Xu, Xue Gao, Lupei Zhang, Junya Li, Huijiang Gao

**Affiliations:** Institute of Animal Sciences, Chinese Academy of Agricultural Sciences, Beijing, China

**Keywords:** ensemble learning, stacking, genomic prediction, machine learning, prediction accuracy

## Abstract

Machine learning (ML) is perhaps the most useful tool for the interpretation of large genomic datasets. However, the performance of a single machine learning method in genomic selection (GS) is currently unsatisfactory. To improve the genomic predictions, we constructed a stacking ensemble learning framework (SELF), integrating three machine learning methods, to predict genomic estimated breeding values (GEBVs). The present study evaluated the prediction ability of SELF by analyzing three real datasets, with different genetic architecture; comparing the prediction accuracy of SELF, base learners, genomic best linear unbiased prediction (GBLUP) and BayesB. For each trait, SELF performed better than base learners, which included support vector regression (SVR), kernel ridge regression (KRR) and elastic net (ENET). The prediction accuracy of SELF was, on average, 7.70% higher than GBLUP in three datasets. Except for the milk fat percentage (MFP) traits, of the German Holstein dairy cattle dataset, SELF was more robust than BayesB in all remaining traits. Therefore, we believed that SEFL has the potential to be promoted to estimate GEBVs in other animals and plants.

## Introduction

Genomic selection (GS) was first introduced by Meuwissen et al. (2001), by using whole-genome markers’ information to predict the genomic estimated breeding values (GEBVs). The first application of GS was on dairy cattle, to improve the selection of better performing genotypes and accelerate the genetic gain by shortening the breeding cycles (Hayes et al., 2009a; Crossa et al., 2017; Tong et al., 2020). After more than 10 years of development, GS has been wildly used in livestock and plant breeding programs with high prediction accuracy (Hayes et al., 2009a; Heffner et al., 2009). Moreover, GS has been applied to improve the prediction of complex disease phenotypes using genotype data (De Los Campos et al., 2010; Menden et al., 2013). However, a critical concern in genomic prediction is the prediction accuracy calculated by the Pearson’s correlation between the estimated breeding values and the corrected phenotypes. Therefore, the exploration of more robust genomic prediction methods is a well-identified searched by breeders. In recent years, there was an increasing interest in applying machine learning (ML) to genomic prediction. Machine learning is a computer program which can optimize a performance criterion using training data, making predictions or decisions without being explicitly programmed (Alpaydin, 2020). The excellent predictive ability for complex problems leads ML to be employed in industries requiring high accuracy, e.g., email filtering, face recognition, natural language processing or stock market forecasting. ML has been used in GS and might have the best performance at the interpretation of large-scale genomic data (De Los Campos et al., 2010). González-Camacho et al. (2018) suggested that ML was a valuable alternative to well-known parametric methods for genomic selection. Montesinos-López et al. (2018) also found that the predictions of the multi-trait deep learning model were very competitive with the Bayesian multi-trait and multi-environment model. In another study, Jubair and Domaratzki (2019) estimated GEBVs of Iranian wheat landraces by ensemble learning, obtaining better results with those than with single machine learning. It is possible to clearly identify a trend from the literature that more breeders are applying machine learning methods to estimate GEBVs in genomic prediction.

Currently, the machine learning methods applied in animal and plant breeding tend to mainly include: support vector regression (SVR), random forest (RF), kernel ridge regression (KRR), multi-layer prediction (MLP) and convolutional neural network (CNN) (Gianola et al., 2011; Libbrecht and Noble, 2015; González-Camacho et al., 2018; Zou et al., 2019). Those machine learning methods possess the ability to predict GEBVs by building a complex non-linear model, considering the interaction effects and epistatic effects (Gianola et al., 2011). Nevertheless, the prediction accuracy of those single machine learning methods did not improve much when compared to the traditional genomic prediction methods [GBLUP, ridge regression BLUP (rrBLUP), BayesB, etc.]. Ogutu et al. (2011) compared the prediction accuracy of RF, boosting and support vector machine (SVM) with rrBLUP in a simulated dataset, in which rrBLUP outperformed the three machine learning methods. When comparing the prediction performance of multi-layer prediction and the SVM with the Bayesian threshold genomic best linear unbiased prediction (TGBLUP), the reliability of two machine learning methods was comparable to, and in some cases, outperformed that of TGBLUP (Montesinos-López et al., 2019). Albeit that the achievement of ML in GS has not been fantastic, breeders are confident on this promising tool. Moreover, even currently associated with certain limitations, it outstands from the other common available methods in the performance.

One of the available solutions to further improve the prediction accuracy of ML in GS is to simultaneously integrate several machine learning methods in genomic prediction. Ensemble learning is an ML paradigm where multiple learners are trained to solve the same problem, therefore, the obtained robustness is higher when compared to that using single learner (Thomas, 1997; Polikar, 2006). Stacking, boosting and bagging were the most common integration strategies used on ensemble learning, among which stacking has a powerful prediction capability for complex problems. In other research areas, stacking has been applied to date prediction, protein-protein interaction prediction, credit scoring, cancer detection, etc. (Wang et al., 2011; Wang Y. et al., 2019; Sun and Trevor, 2018; Yi et al., 2020). However, the application of stacking in GS has rarely been reported.

Therefore, the objective of this study was to improve genomic predictions by using a stacking ensemble learning framework (SELF). In the experiment, SVR, KRR, and ENET were selected as the base learner, and the ordinary least squares (OLS) linear regression was chosen as the meta learner to construct the SELF model. Subsequently, we evaluated the SELF model using two animal datasets (Chinese Simmental beef cattle dataset and German Holstein dairy cattle dataset) and a plant dataset (Loblolly pine dataset). To assess the performance of SELF, we compared the prediction accuracy of SELF with the base learners, GBLUP and BayesB. Finally, the 20-fold cross-validation was employed to mitigate the impact of the accidental error.

## Materials and Methods

### Dataset

#### Chinese Simmental Beef Cattle Dataset

The Chinese Simmental beef cattle population included 1,217 individuals; born between 2008 and 2014 in Ulgai, Xilingolia of China, and were slaughtered at 16 to18 months. After slaughtering, the carcass trait was assessed according to the institutional meat purchase specifications for fresh beef guidelines. At the present study, three important economic traits were selected for latter analysis: live weight (LW), carcass weight (CW), and eye muscle area (EMA). The statistics description for each trait included an estimation of component variance, which is presented in Table 1. The entire Chinese Simmental beef cattle population was genotyped by Illumina^®^ BovineHD BeadChip (770K). The quality control criteria of genotype data were as follows: minor allele frequency (MAF) > 0.05, call rate (CR) > 0.95 and *P*-value > 10^–5^ from Hardy-Weinberg equilibrium (HWE). In addition, the fix effects were used to correct the phenotypes of each trait. Among them, age and sex were regarded as a contemporary group; the fattening time and initial weight were regarded as covariates.

**TABLE 1:** Descriptive statistics of the phenotype data used in the genomic prediction.

Dataset	Trait	N^*a*^	*h*^2^	Mean	SD
Beef cattle	LW	1216	0.53	505.26	70.76
	CW	1216	0.44	271.36	45.65
	EMA	1117	0.57	85.21	13.32
Dairy cattle	MY	5024	0.95	370.79	641.60
	MFP	5024	0.94	−0.06	0.28
	SCS	5024	0.88	102.32	11.73
Loblolly pine	HT	861	0.31	20.30	73.31
	CWAL	861	0.27	2.44	27.32
	TS	910	0.37	0.10	1.22

*N^*a*^, number of the animal with phenotypes; *h*2, heritability; SD, standard deviation. LW, live weight; CW, carcass weight; EMA, eye muscle area; MY, milk yield; MFP, milk fat percentage; SCS, somatic cell score; HT, total stem height; CWAL, crown width along the planting beds; TS, tree stiffness.*

#### German Holstein Dairy Cattle Dataset

The dataset of German Holstein dairy cattle consisted of 5,024 bulls with genotypes and phenotypes (Zhang et al., 2015). The genotype data were generated with the Illumina^®^ Bovine SNP50 BeadChip [42,551 single nucleotide polymorphisms (SNPs)]. All of the SNPs met the following standards: HWE *P*-value > 10^–4^, CR > 0.95 and MAF > 0.01 (Yin et al., 2020). Because the dataset used at the present study was not original, all the phenotype data had been standardized (mean = 0, standard deviation = 1). More details about the original dataset can be found at Zhang et al. (2015). For the German Holstein dairy cattle dataset, the statistics description was based on Zhang et al. (2015) and can be found in Table 1. The phenotypes were described by three traits: milk yield (MY), milk fat percentage (MFP) and somatic cell score (SCS). These three traits may represent three genetic architectures of complex traits composed of: (1) one major gene and a large number of small effect loci (MFP), (2) few moderate effect loci and many small effect loci (MY), and (3) many loci with small effects (SCS), respectively (Zhang et al., 2015; Yin et al., 2020).

#### Loblolly Pine Dataset

The Loblolly pine dataset comprised 951 individuals from 61 families, having 17 traits systemically recorded from each individual (Resende et al., 2012). For the original dataset, all the individuals were genotyped with an Illumina^®^ Iminium assay (7216 SNPs) (Zhang et al., 2015). After quality control, the genotypes contained 4,853 polymorphic SNPs, which were the same as used by Resende et al. (2012) and Zhang et al. (2015). The phenotypes that were used were a subset of the original phenotype data. Within the traits selected, i.e., growth traits (total stem height, HT), development traits (crown width along the planting beds, CWAL) and wood quality traits (tree stiffness, TS), only one trait was chosen to implement prediction models, respectively. The statistics description for the Loblolly pine dataset is shown in Table 1.

### Stacking

**FIGURE 1:**
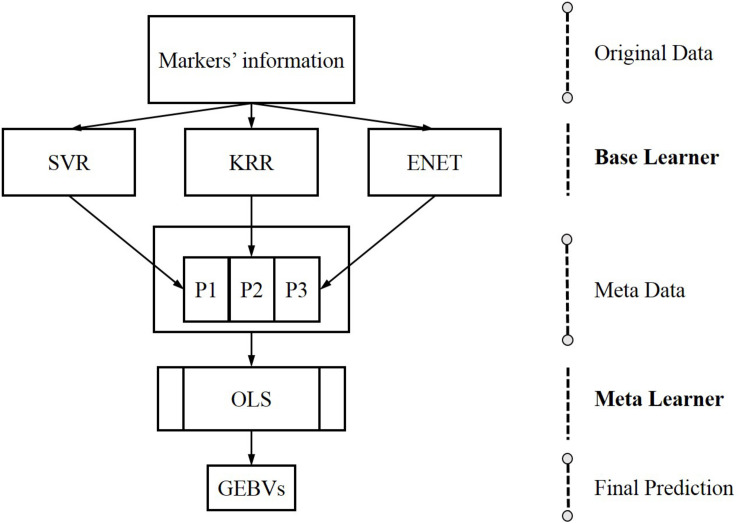
The data flow of stacking ensemble learning framework for genomic prediction, from original data to the base learners, creating metadata for the meta-learner. G, genotypes derived genomic relationship matrix; SVR, support vector regression; KRR, kernel ridge regression; ENET, elastic net; OLS, ordinary least squares linear regression.

Stacking is a form of meta-learning which can yield impressive results by designing novel deep learning architectures (Kyriakides and Margaritis, 2019). The core idea of stacking is using the base learners to generate metadata for the inputs and then utilize another learner, generally called the meta-learner, to process metadata. Base learners are usually called level 0 learners, the meta learners are called level 1 learners and the meta learners stacked on the based learners are the so-called stacking (Kyriakides and Margaritis, 2019). In genomic prediction, the SELF is performed in two steps: firstly, a series of single machine learning methods are trained to generate metadata using markers’ information; secondly, a meta learner are trained to predict GEBVs using metadata. The data flow of SELF for genomic prediction is shown in Figure 1.

The base learners employed to construct SELF at present study, involved SVR, KRR and ENET. SVR and KRR construct a non-linear model to predict GEBVs and ENET estimate the GEBVs by building a linear regression. It is important to highlight that the meta learner should be a relatively simple ML algorithm to (1) avoid overfitting and (2) possess the ability to handle correlated inputs with no assumptions about the independence of features. These two factors will be important because the inputs of meta-learner will be highly correlated (Kyriakides and Margaritis, 2019). Taking into account the above requirements, the OLS linear regression was chosen as the meta-learner in the SELF. During the SELF model training, the genotypes were not taken as the direct inputs, instead, it were replaced by the genomic relationship matrix derived from genotypes (Gianola et al., 2011). Although this might reduce the prediction accuracy of a single machine learning method, it would significantly reduce the time and the memory required for computation. In theory, the calculation time of SELF will be equivalent to five times of that by a single machine learning method, as five-fold cross-validation was used to generate metadata. It is important to highlight that if the same steps of previous studies were used to apply the genotypes as the inputs, the computation time of SELF would be unacceptable. Finally, SELF was run in Python (V3.7) with the help of *sklearn* (V0.22) package. The genomic relationship matrix G was calculated as described by VanRaden (2008):

$$\text{G}=\frac{M\,M^{\prime }}{\sum_{l=1}^{m}2\,p_{j}\,q_{j}}$$

where M is a n × m matrix (n is the number of individuals, *m* is the number of markers) and elements of column *j* in M are 0−2*p*_*j*_, 1−2*p*_*j*_ and 2−2*p*_*j*_ for genotypes A_1_A_1_, A_1_A_2_ and A_2_A_2_; *q*_*j*_ is allele frequency A_1_ at locus *j*, *p*_*j*_ is allele frequency A_2_ at locus *j*th.

### Support Vector Regression

Support vector machine (SVM) is grounded in statistical learning theory. SVR is an application of SVM for regression. SVR utilizes a linear or non-linear kernel function to map the original space to a higher dimensional feature space (Müller and Guido, 2016; Li, 2019). Therefore we built a linear prediction model on feature space. The SVR problem was formalized as:

$$\min_{w,b}\frac{1}{2}\,w^{2}+C\,\sum_{i=1}^{m}L_{\varepsilon }\,\left(f\,\left(x_{i}\right)-y_{i}\right)$$

where *C* is the regularization constant, *L*_ε_ is the ε-insensitive loss:

$$L_{\varepsilon }=\left\{ \begin{array}{c} 0\;\mathrm{if}\,z<\varepsilon \\ \left|\text{z}\right|-\varepsilon ,\mathrm{otherwise} \end{array}\right.$$

where k(*x*_*i*_,*x*_*j*_) = ϕ(*x*_*i*_)^*T*^ϕ(*x*_*j*_) z = *f*(*x*_*i*_) − *y*_*i*_. Through a series of optimization processes, the SVR can be written as:

$$f\left(x\right)=\sum_{i=1}^{m}\left({\overset{⌢}{\alpha }}_{i}-\alpha_{i}\right)k\left(x,x_{i}\right)+b$$

where k(*x*_*i*_,*x*_*j*_) = ϕ(*x*_*i*_)^*T*^ϕ(*x*_*j*_) is the kernel function. In this study, the Gaussian kernel was used to map original data and the most suitable parameters of C and ε for each trait were determined by grid search. The function *SVR* in *sklearn* package (V 0.22) was used to implement SVR methods.

### Kernel Ridge Regression

The difference between KRR and ridge regression is that KRR exploits the kernel trick to define a higher dimensional feature space and then builds the ridge regression model in feature space (Douak et al., 2013; He et al., 2014; Exterkate et al., 2016). For KRR, the final prediction function can be written as the following:

$$f\,\left(x\right)=k^{\prime }\,{\left(K+\lambda \,I\right)}^{-1}\,y$$

where *K* is the so-called gram matrix with entries*K*_*ij*_ = ϕ(*x*_*i*_)⋅ϕ(*x*_*j*_), *k* is a vector with entries *k*_*i*_ = ϕ(*x*)⋅ϕ(*x*_*i*_ = *k*(*x*,*x*_*i*_) with*i* = 1,2,3,…,n, n is the number of training samples; *I* is the identity matrix, λis the ridge parameter. In this study, the kernel was used to transform input data that was selected by the grid search method.

### Elastic Net

Elastic net is a linear regression model trained with both *l*_*1*_ and *l*_*2*_-norm regularization of the coefficients. This combination leads to the ENET, presenting similar advantages when compared to Lasso and ridge regression simultaneously. Thus, ENET can learn a sparse model where few of the weights are non-zero and maintaining the regularization properties (Pedregosa et al., 2011). The progress of training the ENET model can be seen as an optimization process for:

for this study, *X c*is a matrix of the training section of G matrix, ω is the vector of weights, α and ρ are the parameters that determined by grid search.

### Genomic Best Linear Unbiased Prediction

The basic GBLUP method was built by the following equation (VanRaden, 2008; Hayes et al., 2009b):

y=1⁢μ+Z⁢g+e

where *y* is the vector of the correct phenotype, *μ* is the overall mean, *1* is a vector of ones, Z is a design matrix that allocates records to breeding values, *g* is a vector of genomic breeding values, *e* is a vector of residuals. Random residuals were assumed that $e∼N\,\left(0,I\,\sigma_{e}^{2}\right)$ where $\sigma_{e}^{2}$ is the residual variance, *I* is an identity matrix. *g* assumed that $g∼N\,\left(0,G\,\sigma_{g}^{2}\right)$ where $\sigma_{g}^{2}$ is the additive genetic variance, and *G* is the marker-based genomic relationship matrix. To implement GBLUP, we used the *mixed.solve* function of *rrBLUP* package in the R V3.5.

### BayesB

BayesB assumed *a priori* that many markers have no effects, while some have an effect attributed to gamma or exponential distribution (Meuwissen et al., 2009). The formula of BayesB can be written as the following:

$$\text{y}=\sum_{j=1}^{p}m_{j}\,\alpha_{j}+e$$

where y is a vector of phenotypes; *m*_*j*_is the *j*th maker; α_*j*_is the effect of the *j*th maker and$\alpha_{j}∼N\,\left(0,\,\sigma_{\alpha_{j}}^{2}\right)$. The variance of α_*j*_ is assigned an informative before showing the presence (with the probability of 1−π) and absence (with the probability of π) of the marker *j*. The π was determined by the experience before building the BayesB model.

### Cross-Validation

The prediction accuracy of the machine learning methods, GBLUP and BayesB was evaluated with K-fold cross-validation (CV). Each dataset under study was randomly divided into twenty folds by the 20-fold cross-validation. Each fold would be the testing set and the remaining nineteen folds were grouped into the training set. The training set was used to teach the SELF model how to predict the GEBVs of individuals in the testing set. The accuracy obtained and shown in the result section was the mean of prediction accuracy of each testing set which was measured as the Pearson correlation between the corrected phenotypes (*y*) and predicted GEBV (*y*_*pre*_) using the formula

$$r=\frac{c\,o\,v\,\left(y,y_{p\,r\,e}\right)}{\sqrt{v\,a\,r\,\left(y\right)*v\,a\,r\,\left(y_{p\,r\,e}\right)}}$$

## Results

### Comparison Between the Prediction Accuracy of Base Learners, GBLUP and BayesB

Firstly, we described the prediction accuracy of base learners, GBLUP and BayesB for three datasets, as shown in Table 2. BayesB and KRR outperformed other methods in three traits, showing the best predictive power. The prediction accuracy of GBLUP and ENET was higher than that of other methods in two traits. The prediction performance of SVR was the worst, and the prediction accuracy of SVR was always lower than that of the other methods. For base learners, the prediction accuracy of KRR was the highest. The prediction accuracy gap between these methods was not significant, however, the ability to estimate the GEBVs was comparable.

**TABLE 2:** Prediction accuracy of SVR, KRR, ENET, GBLUP, and BayesB for the three datasets.

Dataset	Trait	SVR	KRR	ENET	GBLUP	BayesB
Beef cattle	LW	0.274 ± 0.022	**0.283 ± 0.019**	0.276 ± 0.018	0.256 ± 0.017	0.265 ± 0.016
	CW	0.307 ± 0.016	**0.315 ± 0.015**	**0.315 ± 0.017**	0.292 ± 0.014	0.282 ± 0.012
	EMA	0.280 ± 0.025	0.281 ± 0.022	0.285 ± 0.024	0.292 ± 0.015	0.281 ± 0.015
Dairy cattle	MY	0.764 ± 0.013	**0.781 ± 0.009**	0.762 ± 0.014	0.768 ± 0.006	0.767 ± 0.005
	MFP	0.796 ± 0.012	0.828 ± 0.006	0.797 ± 0.012	0.832 ± 0.003	**0.855 ± 0.003**
	SCS	0.706 ± 0.010	0.751 ± 0.008	0.722 ± 0.019	**0.752 ± 0.006**	0.731 ± 0.003
Loblolly pine	HT	0.340 ± 0.027	0.352 ± 0.011	**0.366 ± 0.014**	0.349 ± 0.012	0.365 ± 0.009
	CWAL	0.352 ± 0.022	0.359 ± 0.018	0.369 ± 0.022	0.384 ± 0.014	**0.400 ± 0.011**
	TS	0.397 ± 0.017	0.407 ± 0.016	0.398 ± 0.015	0.366 ± 0.012	**0.418 ± 0.013**

*The accuracy was calculated by the Pearson’s correlation. LW, live weight; CW, carcass weight; EMA, eye muscle area; MY, milk yield; MFP, milk fat percentage; SCS, somatic cell score; HT, total stem height; CWAL, crown width along the planting beds; TS, tree stiffness. SVR, support vector regression; KRR, kernel ridge regression; ENET, elastic net; GBLUP, genomic best linear unbiased prediction. The bold values mean the highest prediction accuracy for each trait.*

### Comparison Between the Prediction Accuracy of SELF and Base Learners

**FIGURE 2:**
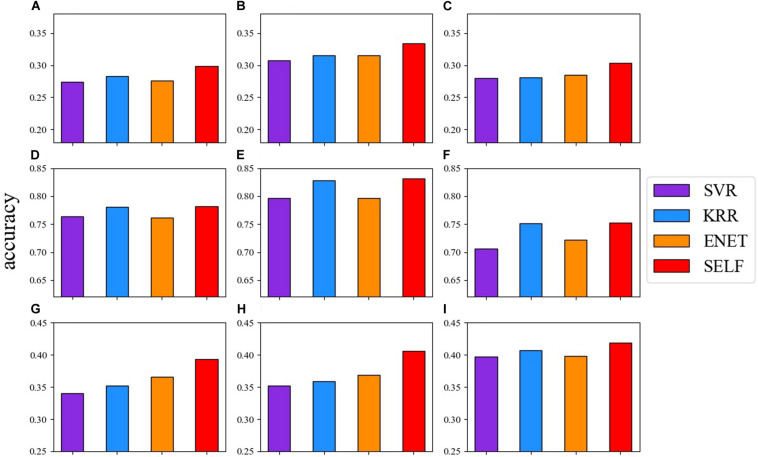
Comparison of the prediction accuracy among: SVR (blue violet), KRR (dodger blue), ENET (dark orange) and SELF for nine traits. **(A)** live weight; **(B)** carcass weight; **(C)** eye muscle area; **(D)** milk yield; **(E)** milk fat percentage; **(F)** somatic cell score; **(G)** total stem height; **(H)** crown width along the planting beds; **(I)** tree stiffness.

Figure 2 shows the comparison between the prediction accuracy of the base learners and SELF for nine traits. The red one represents the prediction accuracy of SELF. SELF performed better than all the other base learners for each trait. Particularly for CWAL, HT, and EMA, the prediction accuracy of SELF was improved by 9.97, 7.36, and 6.40%, respectively, when compared to the highest prediction accuracy of base learners. Among the three base learners, the prediction ability of KRR was comparable to SELF in German Holstein dairy cattle dataset.

### Comparison Between the Prediction Accuracy of SELF, GBLUP and BayesB

**FIGURE 3:**
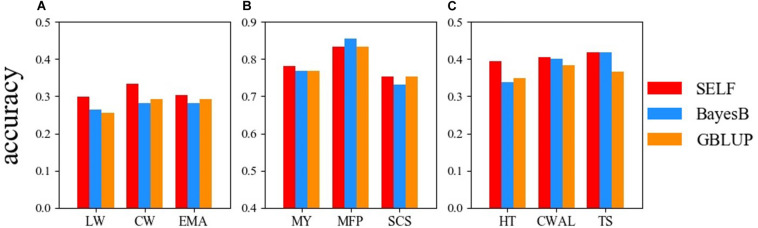
Comparison of the prediction accuracy among: SELF (red), GBLUP (dodger blue) and BayesB (dark orange) for three datasets. **(A)** Chinese Simmental beef cattle dataset; **(B)** German Holstein dairy cattle dataset; **(C)** Loblolly pine dataset. LW, live weight; CW, carcass weight; EMA, eye muscle area; MY, milk yield; MFP, milk fat percentage; SCS, somatic cell score; HT, total stem height; CWAL, crown width along the planting beds; TS, tree stiffness. GBLUP, genomic best linear unbiased prediction; SELF, a stacking ensemble learning framework.

Figure 3 demonstrates the prediction accuracy of GBLUP, BayesB and SELF for the three datasets. For the Chinese Simmental beef cattle dataset, the prediction accuracy of SELF was higher than GBLUP and BayesB, showing an average improvement of 11.68% from SELF to GBLUP. For the German Holstein daily cattle, except for the trait of MFP, SELF performed better than BayesB and GBLUP. For the Loblolly pine dataset, SELF predicted GEBVs more accurately than GBLUP and BayesB, showing an improvement of 14.18% for TS, when compared with GBLUP. Comparing the prediction accuracy between SELF and GBLUP, the average prediction accuracy of SELF was increased by 7.70% in nine traits.

## Discussion

The previous large number of studies had tried to apply single machine learning methods into genomic prediction (Long et al., 2011; Jubair and Domaratzki, 2019; Montesinos-López et al., 2019; Lenz et al., 2020). However, the single machine learning methods applicatied in most of the previous studies, only performed well on certain traits (Long et al., 2011; Ogutu et al., 2011; González-Camacho et al., 2018; Montesinos-López et al., 2019). Therefore, the present study proposed a new strategy to utilize machine learning methods in genomic prediction by using a stacking ensemble learning framework integrating three machine learning methods to predict GEBVs simultaneously. To examine the prediction ability of SELF, we compared the prediction accuracy of SELF with GBLUP and BayesB in animal and plant datasets with a variety of genetic architecture. Considering the computation time and that overfitting was employed, the genotypes derived relationship matrix as the inputs rather than using the genotypes directly (Gianola et al., 2011).

### The Prediction Accuracy of Base Learners, GBLUP, and BayesB

Using GBLUP and BayesB to predict GEBV for the three dataset had been reported early which provided a reference for verifying our results. Therefore, this study compared the prediction accuracy of GBLUP and BayesB with the prediction accuracy obtained from Wang X. et al. (2019), Zhang et al. (2015), and Resende et al. (2012). Wang X. et al. (2019) compared GBLUP with BayesB in the Chinese Simmental beef cattle dataset. Zhang et al. (2015) and Resende et al. (2012) compared the prediction accuracy of different methods on the German Holstein dairy cattle dataset and the Loblolly pine dataset, respectively. Overall, the results were consistent. Since the method was slightly different from that was used in the previous studies, the accuracy differed in individual traits. Although, the application of a single machine learning method to estimate GEBVs on the three datasets has not been reported, the vast majority of studies has compared the prediction accuracy of the single machine learning method with GBLUP or Bayesian family methods on other populations. Therefore, it provided a practical reference when evaluating the performance of single machine learning methods. The results of Ghafouri-Kesbi et al. (2017) and Long et al. (2011) indicated that GBLUP presented better prediction accuracy when compared to SVR and RF. Furthermore, in most cases, the performance of SVR with Gaussian kernel was comparable to that of Bayesian Lasso (Long et al., 2011; Ghafouri-Kesbi et al., 2017). Similar to previously reported studies, the results from the present study also confirmed that single machine learning did not perform significantly better than GBLUP and Bayes methods.

### Excellent Predictive Performance of SELF

**FIGURE 4:**
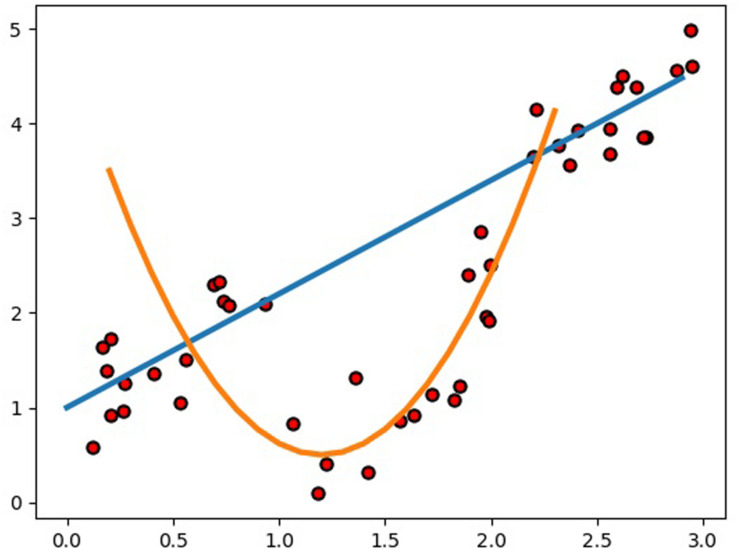
Example of how an integrated model using a stacked ensemble with linear and non-linear regression can significantly outperform either a single linear or non-linear model.

Compared to GBLUP, the average prediction accuracy of SELF was increased by 7.70% for the nine traits, which is significant for animal and plant breeding. Particularly for the beef cattle with a longer generation intervals, such considerable prediction accuracy improvement will greatly accelerate the genetic gain. Actually, it is very difficult to build a SELF model to predict a specific problem with higher accuracy, since the composition of SELF model is so flexible. Therefore, the present study referred to previous studies that using machine learning methods to estimated GEBVs, and combined with our experience to select the candidate base learner. Besides, a single-layer framework or multi-layer framework also should be premeditated carefully when constructing frameworks. Considering the overfitting always accompanied by the machine learning methods in GS and the calculating time of SELF, we determined a single layer stacking framework. Before constructing the model of SELF, RF, SVR, KRR, and ENET were chosen as the candidates for base learners, in which RF and SVR had been performed to predict GEBV in previous studies (Long et al., 2011; Ogutu et al., 2011; González-Recio et al., 2014; Libbrecht and Noble, 2015; Ghafouri-Kesbi et al., 2017). Although the utilization of KRR in genomic prediction had been rarely reported, it was frequently utilized to classification and regression task for other research areas (Douak et al., 2013; Avron et al., 2017; Chang et al., 2017; Naik et al., 2018). In addition, ENET was chosen to achieve more diversification of SELF model due to the reason that SVR, RF, and KRR predicted GEBV by building a non-linear model and ENET was a liner model (Wang Y. et al., 2019). Subsequently to the prediction of GEBVs using four base learners, we decided to exclude RF from the SELF, because RF greatly increased the computation time of SELF. Consequently, the final SELF model was constructed by SVR, KRR and ENET, in which the base learners were used to build different types of models to estimate the GEBVs. Generally, it was reasonable to employ different learning algorithms to explore the relationship between the feature and the target variable (Kyriakides and Margaritis, 2019). For the regression example (Figure 4), we used a stacked ensemble with linear and non-linear regression, showing the possibility to significantly outperform either a single linear or non-linear model. Even though we directly utilized the best prediction of the linear and non-linear models as the outputs of the integrated model without stacking, the performance of the integrated model was greatly improved. Therefore, the constructed SELF could learn more characteristics in different aspects of the input data, and it performed better than either of the base learners.

Besides, the form of input data in this study might be another momentous reason contributed to the higher prediction accuracy of SELF model. The majority of published studies directly employed genotypes as the inputs of machine learning methods. Nevertheless, the number of markers was considerably larger than the number of individuals. In this case, if we used genotypes with no transformed, the number of variables in the prediction model would be an astronomical figure compared to group size. Despite that single machine learning methods were able to solve the problem of “big P and small N,” stronger overfitting was inevitable, which also decreased the prediction accuracy of the SELF. The application of genomic relationship matrix as the input data was completely different, as the genomic relationship matrix was a n × n matrix, whose size is determined by the group sizen. Therefore, the number of variables in the prediction model would be consistent with the number of individuals. Although it might reduce the prediction accuracy of the base learners, it simultaneously and dramatically reduces the risk of overfitting, which potentially improves the prediction accuracy of the SELF.

## Conclusion

The present study proposes a stacking ensemble learning framework integrating SVR, KRR, and ENET to predict GEBVs. The excellent performance of SELF in a variety of genetic architecture datasets indicates that SELF possesses a significant potential to improve genomic predictions in other animal and plant populations.

## Data Availability Statement

Chinese Simmental Beef Cattle dataset: Data is available from the Dryad Digital Repository: 10.5061/dryad.4qc06. German Holstein dairy cattle dataset: Data can be obtained at: https://www.g3journal.org/content/5/4/615.supplemental. Loblolly pine dataset: The quality-controlled genotypes can be gotten at: https://www.genetics.org/highwire/filestream/412827/field_highwire_adjunct_files/1/FileS1.zip and the complete phenotypes at: https://www.genetics.org/highwire/filestream/412827/field_highwire_adjunct_files/4/FileS4.xlsx.

## Ethics Statement

The animal study was reviewed and approved by the Science Research Department of the Institute of Animal Science, Chinese Academy of Agricultural Sciences (CAAS), Beijing, China (approval number: RNL09/07).

## Author Contributions

HG and JL conceived and designed the study. ML and BA performed statistical analyses and wrote the manuscript. ML, JM, and XW wrote the code. TC, BA, XD, LD, and JM participated in data analyses. LZ, LX, and XG participated in the design of the study and contributed to acquisition of data. All authors read and approved the final manuscript.

## Conflict of Interest

The authors declare that the research was conducted in the absence of any commercial or financial relationships that could be construed as a potential conflict of interest.
