# Supplementary material for: A Stacking Ensemble Learning Framework for Genomic Prediction
Source: Front Genet. 2021 Mar 4;12:600040. doi: 10.3389/fgene.2021.600040 (PMC7969712; doi:10.3389/fgene.2021.600040)

Table 1 P-values of the Hotelling's T-squared test and the Student's test comparing the prediction accuracy of GBLUP and SELF

Dataset	Trait	GBLUP		SELF		P_t	$P_{T-squared}$
		mean	std	mean	std		
Beef cattle	LW	0.256	0.077	0.299	0.058	0.053	
	CW	0.292	0.066	0.334	0.060	**0.043**	
	EMA	0.292	0.070	0.303	0.070	0.625	
Dairy cattle	MY	0.768	0.028	0.783	0.023	0.093	
	MFP	0.832	0.012	0.832	0.026	0.994	**0.025**
	SCS	0.752	0.028	0.752	0.035	0.374	
Loblolly pine	HT	0.349	0.057	0.393	0.079	**0.022**	
	CWAL	0.384	0.062	0.406	0.070	0.334	
	TS	0.366	0.053	0.418	0.071	**0.014**	

P_t the P-values of Student's test, $P_{T-squared}$ the P-values of Hotelling's T-squared test

LW live weight, CW carcass weight, EMA eye muscle area, MY milk yield, MFP milk fat percentage, SCS somatic cell score, HT total stem height, CWAL crown width along the planting beds, TS tree stiffness

Table 2. Prediction accuracy of base learners and SELF for the three datasets

Dataset	Trait	GBLUP	BayesB	SELF
Beef cattle	LW	0.256±0.017	0.265±0.016	**0.299**±0.013
	CW	0.292±0.014	0.282±0.012	**0.334**±0.013
	EMA	0.292±0.015	0.281±0.015	**0.303**±0.015
Dairy cattle	MY	0.768±0.006	0.767±0.005	**0.783**±0.005
	MFP	0.832±0.003	**0.855**±0.003	0.832±0.006
	SCS	**0.752**±0.006	0.731±0.003	**0.752**±0.008
Loblolly pine	HT	0.349±0.012	0.365±0.009	**0.393**±0.017
	CWAL	0.384±0.014	0.400±0.011	**0.406**±0.015
	TS	0.366±0.012	**0.418**±0.013	**0.418**±0.016

The accuracy was calculated by the Pearson's correlation. LW, live weight; CW, carcass weight; EMA, eye muscle area; MY, milk yield; MFP, milk fat percentage; SCS, somatic cell score; HT,

total stem height; CWAL, crown width along the planting beds; TS, tree stiffness. SVR, support vector regression; KRR, kernel ridge regression; ENET, elastic net; GBLUP, genomic best linear unbiased prediction.

Fig 1. The PCA analysis of Chinese Simmental Beef cattle

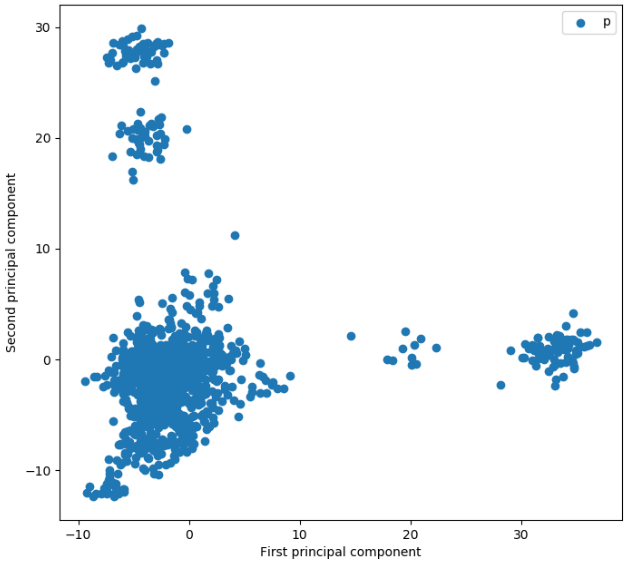

Supplement: Supplementary file 1 [file Data_Sheet_1.PDF]
